# Autoimmune Thyroiditis and Myasthenia Gravis

**DOI:** 10.3389/fendo.2017.00169

**Published:** 2017-07-13

**Authors:** Angela Lopomo, Sonia Berrih-Aknin

**Affiliations:** ^1^Department of Translational Research and New Technologies in Medicine and Surgery, Division of Medical Genetics, University of Pisa, Pisa, Italy; ^2^Sorbonne Universités, UPMC Univ Paris 06, Paris, France; ^3^INSERM U974, Paris, France; ^4^AIM, Institute of Myology, Paris, France

**Keywords:** acetylcholine receptor antibodies, interferon type I, germinal centers, genetics, etiology

## Abstract

Autoimmune diseases (AIDs) are the result of specific immune responses directed against structures of the self. In normal conditions, the molecules recognized as “self” are tolerated by immune system, but when the self-tolerance is lost, the immune system could react against molecules from the body, causing the loss of self-tolerance, and subsequently the onset of AID that differs for organ target and etiology. Autoimmune thyroid disease (ATD) is caused by the development of autoimmunity against thyroid antigens and comprises Hashimoto’s thyroiditis and Graves disease. They are frequently associated with other organ or non-organ specific AIDs, such as myasthenia gravis (MG). In fact, ATD seems to be the most associated pathology to MG. The etiology of both diseases is multifactorial and it is due to genetic and environmental factors, and each of them has specific characteristics. The two pathologies show many commonalities, such as the organ-specificity with a clear pathogenic effect of antibodies, the pathological mechanisms, such as deregulation of the immune system and the implication of the genetic predisposition. They also show some differences, such as the mode of action of the antibodies and therapies. In this review that focuses on ATD and MG, the common features and the differences between the two diseases are discussed.

## Introduction

Autoimmune diseases (AIDs) are the result of specific immune responses directed against structures of the self. Under normal conditions, the immune system is tolerant to molecules recognized as “self” and does not react to antigens expressed in endogenous tissues. If self-tolerance is missing, the immune system could develop an immune response against self-molecules, causing the development of AIDs that include autoimmune thyroid diseases (ATDs) and myasthenia gravis (MG).

The etiology of AIDs is multifactorial and involves genetic and environmental factors. ATDs are endocrine diseases due to an autoimmune reaction against thyroid antigens, in a specific genetic background triggered by exposure to environmental factors (1).

The two main ATDs are Graves disease (GD) and Hashimoto’s thyroiditis (HT) that are characterized by hypothyroidism and thyrotoxicosis, respectively, by the production of thyroid autoantibodies such as thyroid peroxidase (TPO), thyroglobulin (TG), and thyroid-stimulating hormone receptor (TSHR), as well as by lymphocytic infiltration of the thyroid (1). MG is a neuromuscular disorder due to a defective transmission of the nerve impulse to muscles, causing muscle weakness and abnormal fatigability. In most cases, MG is mediated by antibodies targeting the acetylcholine receptor (AChR) while in a minority of patients, the autoantibodies are specific for muscle-specific kinase (MuSK) or agrin receptor LRP4 (low-density lipoprotein receptor-related protein-4). Other targets, such as titin, and ryanodine, have been investigated (2).

## Concomitant Thyroiditis and MG: Epidemiological Features

The prevalence of ATDs is high and estimated to be 5% in the general population (3) while MG is a rare disease with an incidence of 8–10 cases per one million persons/year and a prevalence of 150–250 cases per one million (4) (Table 1). Although ATD is one of the most representative organ-specific autoimmune disorders, it is associated with other autoimmune endocrine failures or non-endocrine diseases (5). Among the non-endocrine diseases, we can mention vitiligo, pernicious anemia, MG, autoimmune gastritis, celiac disease, and hepatitis (6, 7). Interestingly, in these associated diseases, the presence of anti-TPO antibodies is more frequent than the prevalence of ATDs (6).

**Table 1:** Epidemiological and clinical features of patients with autoimmune thyroid diseases and myasthenia gravis (MG).

	Hashimoto’s thyroiditis	Graves’ disease	AChR-MG	MuSK-MG
				
	Hypothyroidism	Hyperthyroidism		
Incidence	About 2%	About 2%	About 0.1%	About 0.01%
Female/male ratio	Around 10	Around 10	Early onset: F > M (ratio around 3)	Around 6
			Late onset: F = M	
Tissue pathology	Damage of the thyroid gland	Enlarged thyroid (diffuse goiter)	Thymic pathologies, hyperplasia among the young patients, and thymoma among the oldest patients	No thymic pathology
Therapy	Levothyroxine (LT4)	Anti-thyroid drugs, radioactive iodine, and surgery	Anticholinesterase drugs, thymectomy, immunosuppressive drugs (azathioprine, corticosteroids)	Corticosteroids
				Rituximab

*The data on this table were collected from the following Ref. (8–12)*.

The aim of this review is to focus on the association between MG and ATDs. The most common diseases coexisting with MG are GD and HT, with a frequency of 7 and 3%, respectively (13). ATDs were diagnosed in 26.8% of MG Polish patients including 4.4% with GD, 9% with HT, and 13.4% with anti-thyroid antibodies (14). In British and German populations, 16% of early-onset MG (EOMG), 9% of late-onset MG, and 17% of thymoma-MG patients had antibodies against TPO or TG (15). About 0.2% of patients affected by ATDs show MG that is much higher than the general incidence of MG (0.01%). MG could be ocular or generalized, even if ATDs are more frequent in the ocular group (16–18). When associated to ATDs, MG shows specific features, such as the young age of onset, mild clinical symptoms, low levels of AChR antibodies, and low frequency of thymic alterations (18–20). These data highlight that the association between MG and ATDs is much more frequent than expected.

## Etiology

The etiology of AIDs is still unknown. Drugs, virus infections, radiation, stress are some of the environmental factors that may be involved in the development of ATDs and MG in susceptible individuals, contributing to the activation of an innate immune response (8, 10, 21, 22).

### Factors of Predisposition

Autoimmune thyroid diseases are more common among women than men with a female:male ratio of 5–10:1. There is a difference in prevalence and incidence in the base of geographic area, race, and age. The frequency of anti-thyroid antibodies increases with age, showing a peak ranging from 45 to 55 years. In females, one of the two X chromosomes is inactivated during early embryonic stage (23). The inactivation of the same X chromosome, that occurs in more than 80% of cells, could result in defect in immunological tolerance to X-linked antigens that could lead to autoimmunity. Moreover, fetal microchimerism was observed in blood and thyroid tissues from women with either HT or GD. During pregnancy, the production of maternal regulatory T cells (Treg) early in pregnancy could lead to a decrease in the circulating anti-thyroid antibodies, maintaining a state of tolerance to fetal alloantigens in order to avoid fetus rejection. After birth, anti-thyroid antibodies rebound with a transient increase. The persistence of fetal cells in maternal tissues leads to fetal microchimerism (24).

In MG, instead, the early-onset forms, characterized by the age of onset before 50 years, are more frequent in female than male with a ratio female:male of 3:1. Different studies suggest an important role of estrogens in MG (25), since estrogen receptors are expressed on thymic epithelial cells and on thymocytes (21). The female bias in AIDs could be due to reduced expression by estrogens of AIRE, a transcription factor involved in negative selection, resulting in a decreased quality of autoreactive cells elimination (26).

**Figure 1:**
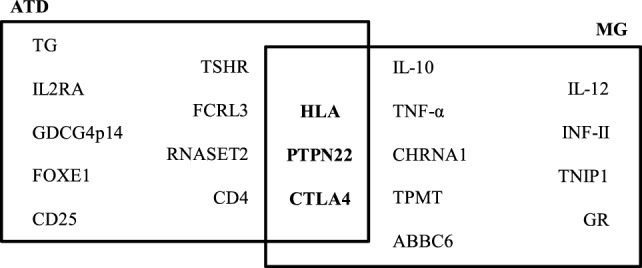
Predisposing genes in autoimmune thyroid diseases (ATDs) and myasthenia gravis (MG). The data on this figure were collected from the following Ref. (28–30).

By case–control studies, and more recent genome-wide association studies, different genes have been associated with the ATDs and MG and the presence of specific autoantibodies. Genes involved in T-cell activation and regulation, such as protein tyrosine phosphatase non-receptor 22 (*PTPN22*), cytotoxic T-lymphocyte antigen-4 (*CTLA4*), and human leukocyte antigens (HLA), are associated with both ATDs and MG. PTPN22 is an intracellular protein tyrosine phosphatase bound to c-src tyrosine kinase, involved in T-cell activation (27); CTLA4 plays a role in inhibiting T-cell signaling, and the HLA is essential for presenting exogenous antigens for recognition by CD4^+^ T-helper cells (28). Other genes have been associated to a single disease, as indicated in Figure 1. Therefore, in both ATDs and MG, factors of predisposition include not only genetic background, but also the potential role of sexual hormones.

### Triggering Factors

Hepatitis C virus (HCV) infection is the most associated to ATDs both in adults and children, in fact, infected HCV patients show dysfunctions in the thyroid (10, 31). In patients with chronic hepatitis C (CHC), the thyroid disorders are characterized by an increased risk of ATD and hypothyroidism in females, elevated levels of anti-TPO antibodies, and by papillary thyroid cancer risk (32, 33). The hypothesized mechanism is that HCV envelope protein E2 induces strong inflammatory responses in human thyrocytes, resulting in the production of interleukin (IL)-8, IL-6, and tumor necrosis factor-α (TNF-α). The E2 protein also induces the upregulation of molecules involved in innate immune pathways (34). Also human herpes virus-6 (HHV-6) infection is associated with ATD onset, in fact, a high level of HHV-6 activation marker was found in thyroid tissue of patients with ATD (35). In myasthenic patients, the existence of a chronic inflammatory state in the thymus could alter innate immune responses leading to self-tolerance failure (36–39). The inflammatory state could be due to persistent viral replications, in fact, Epstein Barr virus (EBV), cytomegalovirus, human foamy virus, and Nile virus were found to be associated to MG (40, 41). Pathogen infections could play a role in AIDs through dysregulation of toll-like receptor-mediated innate immune responses, which can result in altered innate immune responses and long-term inflammation, rendering the thymus vulnerable to auto-sensitization (40, 41). EBV is one of the main candidates suspected to play a role in MG initiation, since it is able to promote B-cell abnormal activation and survival, and to disrupt critical B-cell tolerance checkpoints (40, 42–44).

Recent data have confirmed a strong association between ATD development and interferon (IFN)-α therapy in patients with CHC. About 40% of CHC patients acquire thyroid disorders while receiving IFN-α. IFN-induced thyroiditis is visible as clinical thyroiditis in about 15% of HCV patients receiving IFN-α and subclinical thyroiditis in up to 40% of patients (45). Moreover, it was observed that the generation of anti-thyroid antibodies tends to continue also after IFN therapy (30). IFN-α could induce thyroiditis by both direct toxic effects on the thyroid and by immune recruitment mechanisms (46).

Interestingly, IFN-I therapies can also prime the development of MG (47). IFN-I, especially IFN-β, could play a central role in the thymic follicular hyperplasia of MG patients by inducing high expression of α-AChR and of CXCL13 chemokine in thymic epithelial cells, and of the chemokine CCL21 in endothelial lymphatic cells, two chemokines involved in the abnormal recruitment of B cells in EOMG thymuses. IFN-β also increases B-cell activating factor expression, which promotes the development of autoreactive B cells (48). Also, IFN-β overexpression in MG thymus can mediate the effects of dsRNA activation and causes α-AChR subunit overexpression, suggesting that IFN-β can play a central role in MG development (36).

Other drugs can induce AIDs, including immunomodulatory agents used to treat melanoma, such as monoclonal antibodies inhibiting the immune checkpoint pathways, as CTLA4 and programmed cell death protein 1 (PD-1), two-cell surface receptors on T cells which down-regulate immune response (49). Ipilimumab is a human immunostimulatory antibody targeting CTLA4 that can cause thyroiditis and/or hypothyroidism in 6% of cases after several cycles. Pembrolizumab and nivolumab act against PD-1 and, if combined with ipilimumab to inhibit both CLT4 and PD-1, show a stronger effect with thyroiditis in 22% of cases (50).

## Differential and Common Features in ATDs and MG

### Antibodies

Both MG and ATD diseases are organ specific and antibody-mediated, and both kinds of disorders combine many different pathologies. Patients with ATDs have antibodies against proteins of the thyroid, but the characteristics of the disease differ according to the autoantigen. Patients with HT have serum antibodies reacting with TG, TPO, while patients with GD have antibodies against the receptor of TSH (51) (Table 2).

**Table 2:** Physiopathological features of patients with autoimmune thyroid diseases and myasthenia gravis (MG).

		Hashimoto’s thyroiditis	Graves’ disease	AChR-MG	MuSK-MG
Humoral immunity	Target of the autoantibodies	TG (20–50%), TPO (90–95%)	TSHR	AChR	MuSK
	Mechanism of the Abies	Thyroid destruction by cytotoxic cells, death receptors, and impairment of thyroid hormone production	Overactivation of the gland: thyroid stimulatory, blocking, and neutral Abies	AChR blocking, internalization, and degradation	Disruption of neuromuscular junction and inhibition of the retrograde signaling
	Role of complement	Yes	Yes	Yes	No

Cellular mechanisms	Infiltration of the target organ	+++ Thyroid	+ Thyroid, but not destruction	Neg in the muscle	Neg in the muscle
				+++ in the thymus	Neg in the thymus
	Ectopic GC	Yes (thyroid)	Yes (thyroid)	Yes (thymus)	No
	T-cell involvement	Th1, Th17	Th2, Th17	Th1, Th2, Th17	Th1, Th17
	Role of epithelial cells	Overproduction of pro-inflammatory cytokines and chemokines by thyroid epithelial cells		Overproduction of pro-inflammatory cytokines and chemokines by thymic epithelial cells	Unknown
	Regulatory B cells	Normal B10 number	Normal B10 number	Decreased B10 cell number	Decreased B10 cell number

*The data on this table were collected from the following Ref. (1, 52–58)*.

Myasthenia gravis is due to antibodies against the neuromuscular junction (59). Similarly to thyroiditis, in MG, several antigens are the targets of the autoantibodies, and the disease features depend upon the nature of the antibodies. Patients with anti-AChR, but not with anti-MuSK antibodies, have thymic pathologies, hyperplasia among the young patients, and thymoma among the oldest patients (60).

Interestingly, in both MG and ATDs, some forms of the disease are IgG4 dependent, an Ig subclass that does not bind to the complement. In MG, anti-MuSK antibodies are IgG4 (61). In ATDs, several subcategories of IgG4-mediated diseases have been identified including a fibrosing variant of HT, IgG4-related HT, and GD with elevated IgG4 levels (62). These IgG4 diseases share common mechanisms that involve the mechanical interference of extracellular ligand–receptor interactions by the IgG4 antibodies (63).

The mechanisms of action of the antibodies are quite different in MG and ATD, likely due to the nature of the target antigen and its localization. In HT, together with cytotoxic cells, the antibodies contribute to the destruction of the thyroid, leading to hypothyroidism (Table 2). In the case of GD, the antibodies against TSHR could be stimulatory, blocking or neutral; when the stimulating antibodies predominate, clinical features become obvious (56). Thus, the antibodies are functional, able to stimulate or to inhibit the secretion of thyroid hormones. Fluctuating antibody levels can lead to syndromes alternating between hyperthyroidism and hypothyroidism (64). In the case of MG, anti-AChR antibodies induce its degradation dependent upon the complement, and its internalization (2), while anti-MuSK antibodies disrupt the neuromuscular junction and inhibit the retrograde signaling (65, 66). Recent findings suggest that the anti-AChR antibodies could also have a functional effect, by inducing the overproduction of IL-6, a cytokine that plays a role in muscle biology (67). It is not clear yet if this mechanism participates to the pathogenic mechanisms or is a compensatory mechanism.

Most of the autoantibodies have a clinical usefulness. Anti-TPO and anti-TSHR antibodies are relevant for the diagnosis of HT and GD, respectively (30, 68). Anti-TSH receptor antibodies are of interest in GD as they correlate with the disease severity and their levels decrease with therapies (69). However, anti-TPO and anti-TG Abs are not unique to HT patients since these antibodies are detectable in the majority of GD patients (70). In the case of MG, the anti-AChR antibodies are very useful for the diagnosis but not for the follow-up. On the other hand, for the group of patients with anti-MuSK antibodies, monitoring its level is relevant, since it correlates with the clinical course (2).

### Infiltration of the Target Organ and Germinal Centers

Patients with GD can have an infiltration of the thyroid gland, while in the case of HD, the infiltration is severe and accompanied by the destruction of the thyroid (71). Ectopic B-cell follicles are observed in the thyroid gland in HT (72). Autoreactive B cells within these lymphoid follicles were recognized by their ability to bind thyroid antigens (72). In MG, the neuromuscular junctions displays minimal lymphocytic infiltration, while the thymus at least in the young patients includes many infiltrating cells, signs of inflammation, and germinal centers (53). The degree of hyperplasia is related to the level of anti-AChR antibodies (73).

### Immune Dysregulation

In both MG and ATDs, T-cell immune-mediated mechanisms are involved. In ATDs, cellular immunity targeting thyroid antigens is very common (74, 75). This mechanism is also a feature of experimental thyroiditis obtained in animals by injection of thyroid antigen with adjuvants (76). In MG, similar data are observed; in the patients, and in the experimental models of MG, T-cell proliferation using the autoantigen or peptides from the AChR has been shown (77–79).

In addition, inflammatory cells such as Th1 and Th17 were shown to be involved in the different forms of thyroid or myasthenic diseases (Table 2). Th1 cytokines are increased in MG patients and its experimental model (EAMG) and normalized with therapies (80, 81). Th1 cells and their cytokines are required for EAMG development (82), through the production of complement-dependent anti-AChR antibodies that are pathogenic (82, 83). In addition, TNF-α has been shown to contribute to the chronic inflammation observed in the MG thymus (84). In ATDs, Th1 cells recruited in the thyroid may be responsible for increased production of IFN-γ and TNF-α, which in turn stimulates the secretion of the pro-inflammatory chemokine CXCL10 from the thyroid cells, resulting in an amplification feedback loop, which could perpetuate the autoimmune process (1). Th17 cells and IL-17 have an inflammatory and pathogenic role in MG and ATD (85). Interestingly, IL-17 also contributes to B-cell responses. Indeed, mice mutated for IL-17 receptor have reduced humoral responses and germinal center development (86). In MG patients, the seric level of IL-17 is increased (84, 87). In the mouse model, IL-17 deficient mice are resistant to develop MG, and the pathogenic anti-murine AChR antibodies are lower compared with wild-type mice (88). In ATDs, an increased differentiation of Th17 lymphocytes and an enhanced synthesis of Th17 cytokines were shown, mainly in HT (89).

Finally, the defects of immune regulation are a hallmark of AIDs. In both MG and thyroiditis, functional defects of Treg cells have been shown while the cell number is normal (90–92). In addition, there is a shift from Treg cells to Th17 cells, suggesting that Treg/Th17 balance is altered (84, 93). However, in MG, it was also demonstrated that the Teff cells are resistant to suppression (84). To our knowledge, there is no equivalent study in ATDs disorders. The number of B reg cells has been shown to be decreased in MG but not ATDs (55, 58, 94).

Microbiota is essential for immunologic and digestive homeostasis (95) and is involved in many AIDs (52). In animals, the lack of microbiota is associated with reduced intestinal surface areas with shorter villi, changes in mucus layer, and permeability (5, 96), together with reduced B- and T-cell production (97). Interestingly, in humans, a morphological and functional damage of the intestinal barrier was similar in patients bearing type-1 diabetes and with ATD (98). In addition, in hyperthyroid patients, the microbiota composition was shown to be altered (99). This aspect has not yet been investigated in MG.

## Therapies

Although both MG and ATDs are associated with immune system defects, the treatments are different. In the case of MG, treatments include anticholinesterase molecules and immunosuppressive therapies. Among these therapies are conventional immunosuppressant, such as azathioprine, as well as corticosteroids. Recently, monoclonal antibodies against B lymphocytes have proved interesting (100). In the case of thyroid disease, therapies are aimed at regulating thyroid hormone levels (101). The treatment of choice for HT or hypothyroidism is thyroid hormone replacement. The drug is orally administered usually for life.

Surgery is applied in both pathologies. In MG, thymectomy may be proposed when the thymus is hyperplastic or when a thymoma is associated. Recently, a Thymectomy Trial in Non-Thymomatous MG Patients Receiving Prednisone Therapy was conducted in order understand if transsternal thymectomy with prednisone therapy could be more efficient than prednisone alone after 3 years. An improvement of clinical outcomes over a 3-year period in patients with non-thymomatous MG underwent thymectomy was observed (102). In ATDs, thyroid ablation is recommended in GD when the goiter is large, and in HT when a defined thyroid nodule is present (68). It is interesting to note that in both cases the organ that is operated is inflammatory and contains germinal centers with B lymphocytes participating in the pathogenic response.

## Conclusion

In conclusion, MG and ATD share many commonalities. They are both organ-specific AIDs with a clear pathogenic effect of antibodies, although ATDs are 50–100 times more frequent than MG disease.

Interestingly, ocular involvement is observed in both pathologies. The pathological mechanisms high up many commonalities, such as deregulation of the immune system and the implication of predisposing genes, such as HLA and *PTNP22* genes. However, the mode of action of the antibodies is different: if the antibodies in ATDs deregulate the level of the hormones, in the case of MG they reduce the expression of receptors at the motor plate leading to a functional defect of the synapse.

It is interesting to note that certain drugs are capable of inducing MG and ATDs, for example, IFN-I or monoclonal antibodies against immune checkpoints have proved to be inducers of these pathologies.

Finally, therapies in MG and ATDs are different. In ATDs, the use of molecules to regulate the level of the hormones is satisfactory. In MG, anticholinesterases are generally insufficient, and immunosuppressive therapy is very frequently associated.

## Author Contributions

AL and SB-A discussed the content of the review and wrote the manuscript.

## Conflict of Interest Statement

The authors declare that the research was conducted in the absence of any commercial or financial relationships that could be construed as a potential conflict of interest. The reviewer, CR, and handling editor declared their shared affiliation, and the handling editor states that the process nevertheless met the standards of a fair and objective review.
